# Supplementary material for: CUT&Tag for efficient epigenomic profiling of small samples and single cells
Source: Nat Commun. 2019 Apr 29;10:1930. doi: 10.1038/s41467-019-09982-5 (PMC6488672; doi:10.1038/s41467-019-09982-5)
Supplement: Supplementary file 1 — Supplementary Information [file 41467_2019_9982_MOESM1_ESM.pdf]

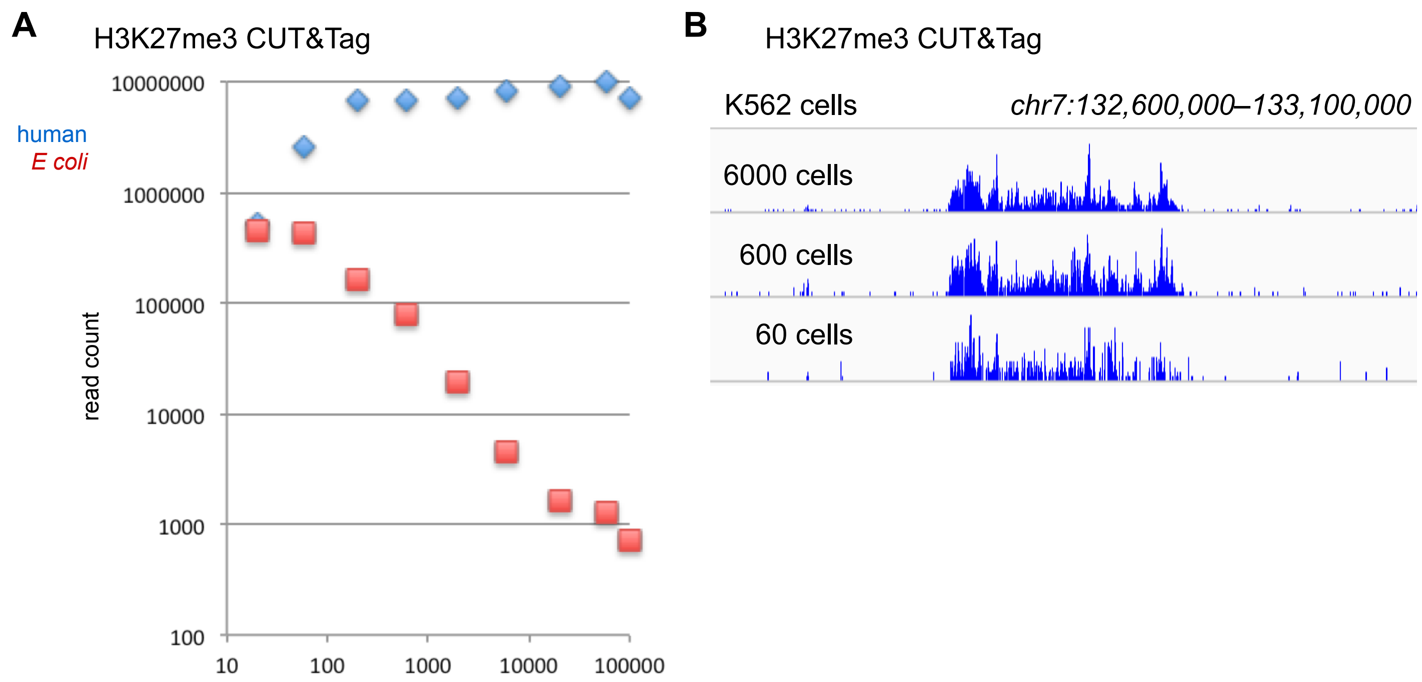

Supplementary Figure 1. *E. coli* carry-through DNA provides a spike-in proxy for CUT&Tag.
(a) Mapping of paired-end reads from a CUT&Tag human cell-dilution experiment to the human and *Escherichia coli* genomes shows that the number of *E. coli* reads increases proportionally with reduced cell numbers over at least 3 orders of magnitude. This indicates that calibration between samples in a treatment series can be achieved for CUT&Tag by dividing the number of reads mapping to the experimental genome by the number of *E. coli* reads, obviating the need for a heterologous spike-in standard.
(b) Chromatin landscapes of the H3K27me3 histone modification in K562 cells from limited cell numbers.

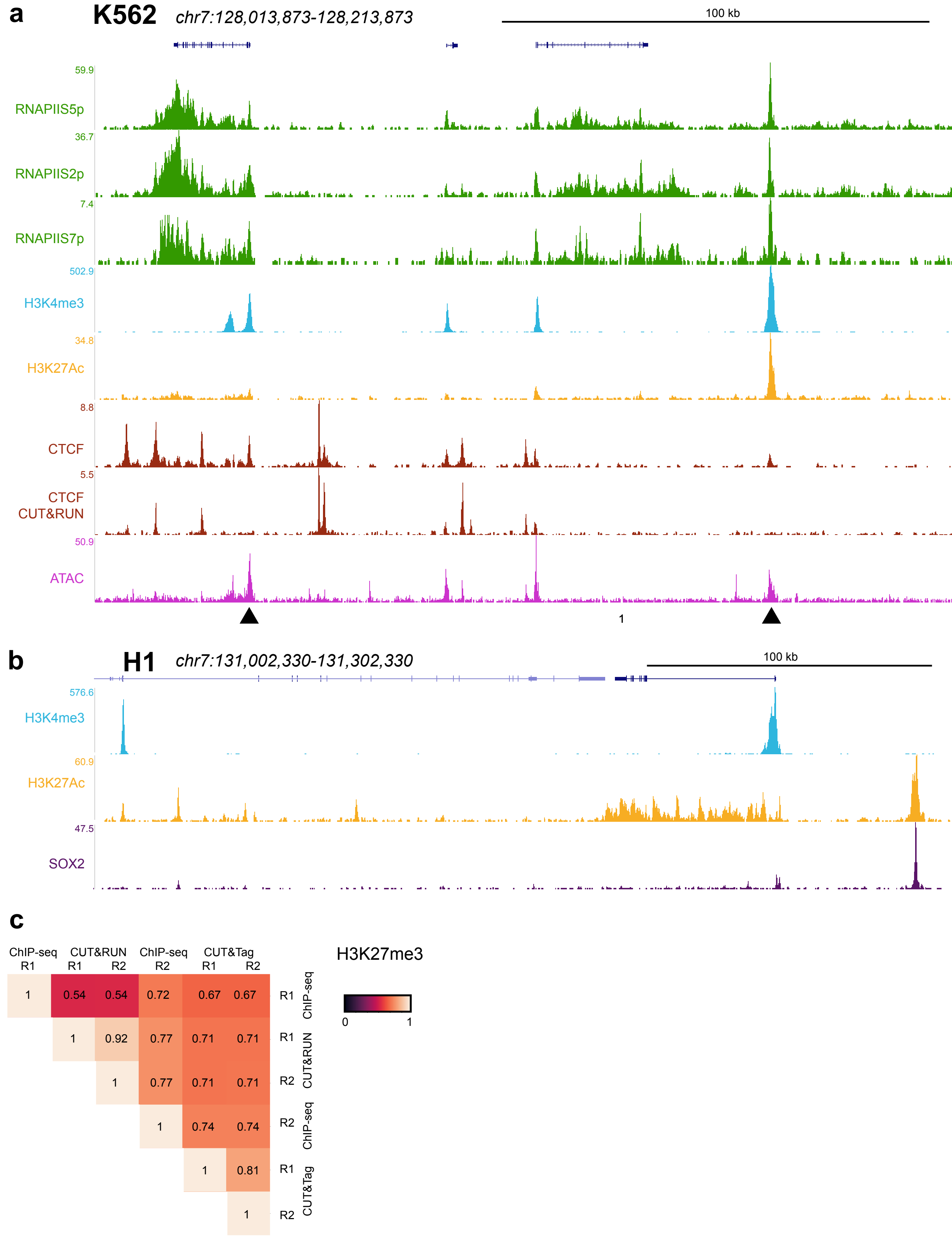
a
K562
chr7:128,013,873-128,213,873
100 kb
59.9
RNAPIIS5p
36.7
RNAPIIS2p
7.4
RNAPIIS7p
502.9
H3K4me3
34.8
H3K27Ac
8.8
CTCF
5.5
CTCF
CUT&RUN
50.9
ATAC
1
b
H1
chr7:131,002,330-131,302,330
100 kb
576.6
H3K4me3
60.9
H3K27Ac
47.5
SOX2
c
ChIP-seq R1
CUT&RUN R1 R2
ChIP-seq R2
CUT&Tag R1 R2
H3K27me3
1 0.54 0.54 0.72 0.67 0.67
1 0.92 0.77 0.71 0.71
1 0.77 0.71 0.71
1 0.74 0.74
1 0.81
1
R1 ChIP-seq
R1 CUT&RUN
R2
R2 ChIP-seq
R1 CUT&Tag
R2
0
1

Supplementary Figure 2. Chromatin profiling by CUT&Tag.

(**a-b**) Additional CUT&Tag profiling landscapes for histone modifications and transcription factors in (**a**) K562 and (**b**) H1 ES cells. The arrowheads indicate hypersensitive (ATAC) sites that appear as CTCF CUT&Tag peaks but are absent from CTCF CUT&RUN profiling.

(**c**) Hierarchically clustered correlation matrix of CUT&Tag, CUT&RUN, and ChIP-seq profiling replicates (R1 and R2) for the H3K27me3 histone modification. The same antibody was used in all experiments. Pearson correlations were calculated using the log_2-transformed values of read counts split into 500 bp bins across the genome.

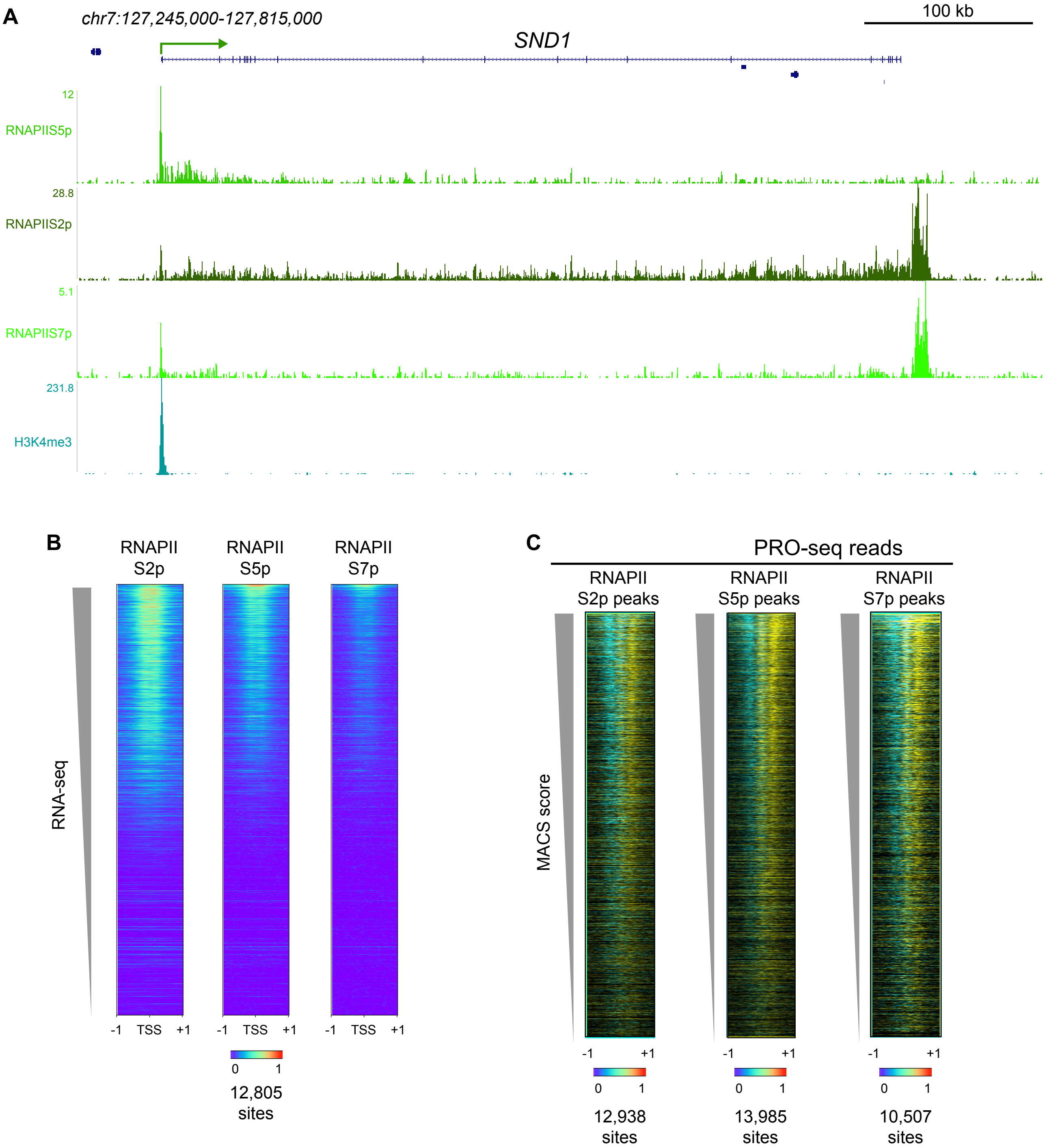
A
chr7:127,245,000-127,815,000
100 kb
SND1
12
RNAPIIS5p
28.8
RNAPIIS2p
5.1
RNAPIIS7p
231.8
H3K4me3
B
RNAPII S2p
RNAPII S5p
RNAPII S7p
RNA-seq
-1 TSS +1
0 1
12,805 sites
C
PRO-seq reads
RNAPII S2p peaks
RNAPII S5p peaks
RNAPII S7p peaks
MACS score
-1 +1
12,938 sites
13,985 sites
10,507 sites

Supplementary Figure 3. Profiling of gene activity by CUT&Tag.
(a) RNAPII CUT&Tag marks the promoters of active genes. Chromatin landscapes of CUT&Tag profiling across the *SND1* gene.
(b) RNAPII CUT&Tag on gene promoters. Annotated genes were ordered by expression as determined by RNA-seq read counts, and RNAPII CUT&Tag reads were plotted for three different RNAPII modifications.
(c) Active RNAPII is enriched at RNAPII CUT&Tag peaks. Peaks were called from RNAPII CUT&Tag for different modifications using MACS2. PRO-seq reads were displayed onto these positions for (+) strand reads (yellow) and (–) strand reads (blue).

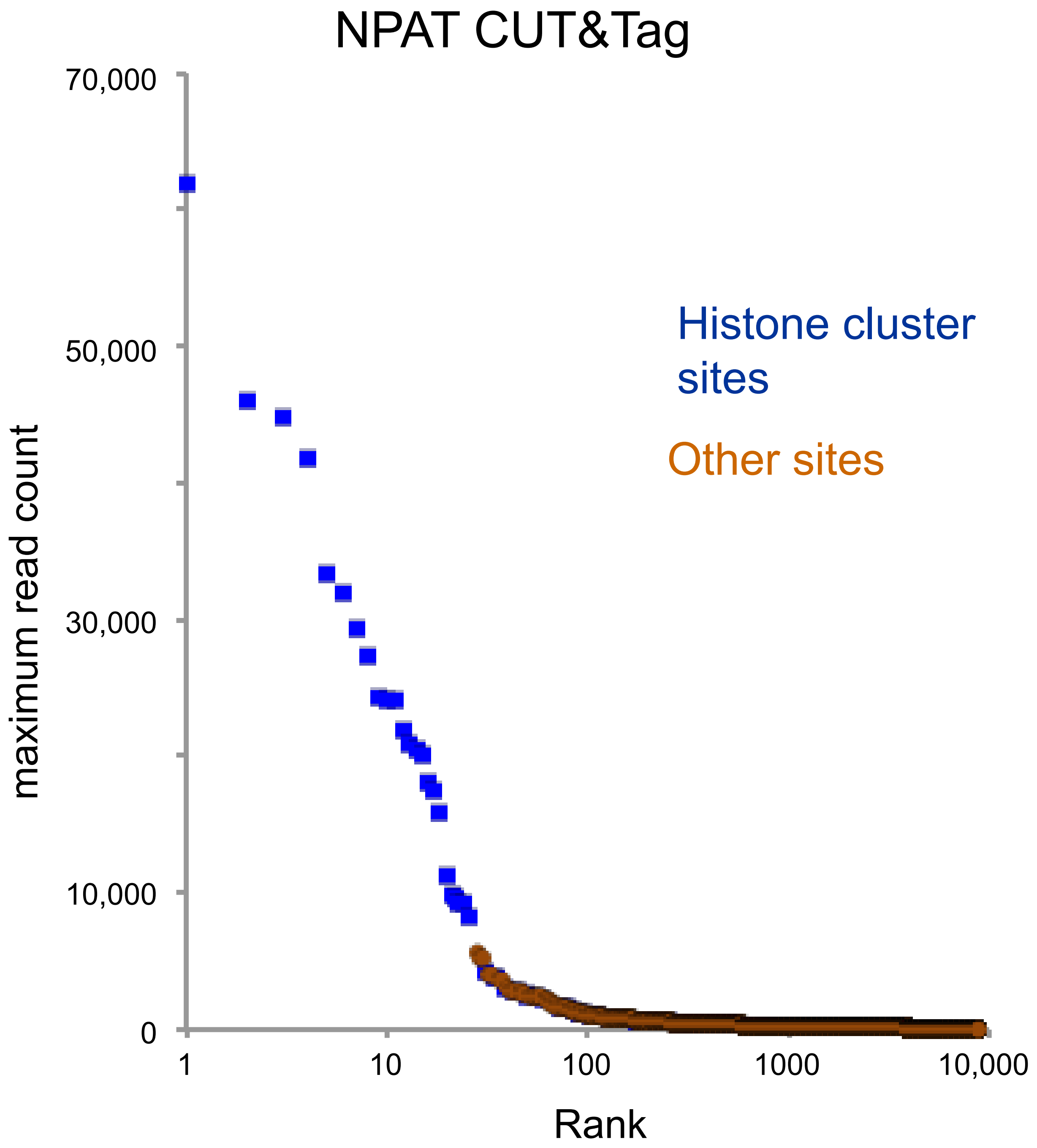

Supplementary Figure 4. Discrimination of NPAT binding sites from accessible sites.
We called 8,689 significant peaks using MACS2 on NPAT CUT&Tag data from K562 cells. The ordered list of sites is plotted against the maximum read count at each site. Sites falling within the histone gene clusters were color-coded blue, and the remaining sites are colored orange.

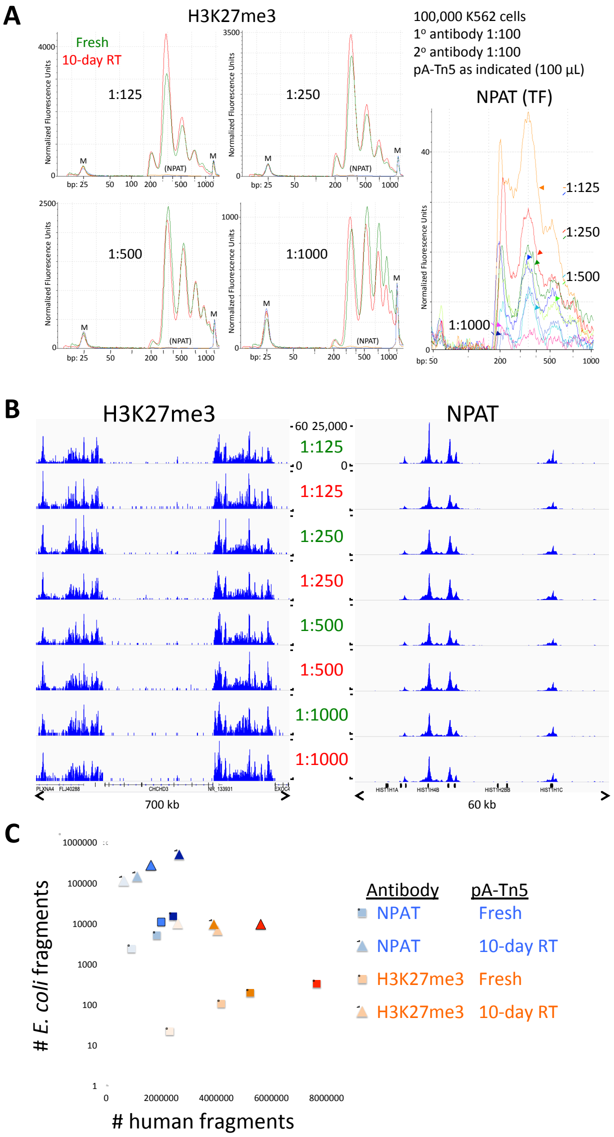

Supplementary Figure 5. pA-Tn5 complex is stable at room temperature.

(**a**) pA-Tn5 in glycerol storage buffer was loaded with adapters and stored at -20°C. A 5 µL aliquot was removed to a PCR tube and held for 10 days at room temperature (RT). CUT&Tag was performed by splitting the samples into 8 50 µL aliquots following the secondary antibody incubation, washing twice in Dig-wash buffer, then incubating 1 hr at RT with serial dilutions of either Fresh or RT pA-Tn5 in Dig-med buffer as indicated. After three Dig-med washes, cells were resuspended in Dig-med + 10 mM $MgCl_2$ and incubated 1 hr 37°C for tagmentation. DNA was extracted and 70% of the total DNA was subjected to 14 PCR cycles. After a single 1.1X Ampure-bead clean-up, DNA was eluted with 25 µL 10 mM Tris-HCl pH 8, and Tapestation D-1000 analysis was performed on a 2 µL sample. Markers (M): lower = 25 bp and upper = 1500 bp. Whereas H3K27me3 is an abundant histone modification, NPAT is a transcription factor (TF) that is specific for the histone loci on human chromosomes 1 and 6, and therefore has very few target sites in the genome.

(**b**) Examples of tracks from serial dilution of pA-Tn5 (50 µL volume). All tracks for each antibody are at the same genome-normalized scale, 0-60 for H3K27me3 around the CHCHD3 locus (Chr7:132,280,000-132,980,000) and 0-25,000 for NPAT around part of a histone cluster (Chr6:26,010,000-26,070,000).

(**c**) *E. coli* carry-over DNA levels increase by 1-2 orders-of-magnitude during 10-day room temperature incubation of pA-Tn5. Counts of human and *E. coli* fragments are shown for the samples in panel B, where shading indicates serial dilutions from 1:125 (dark) to1:1000 (light).

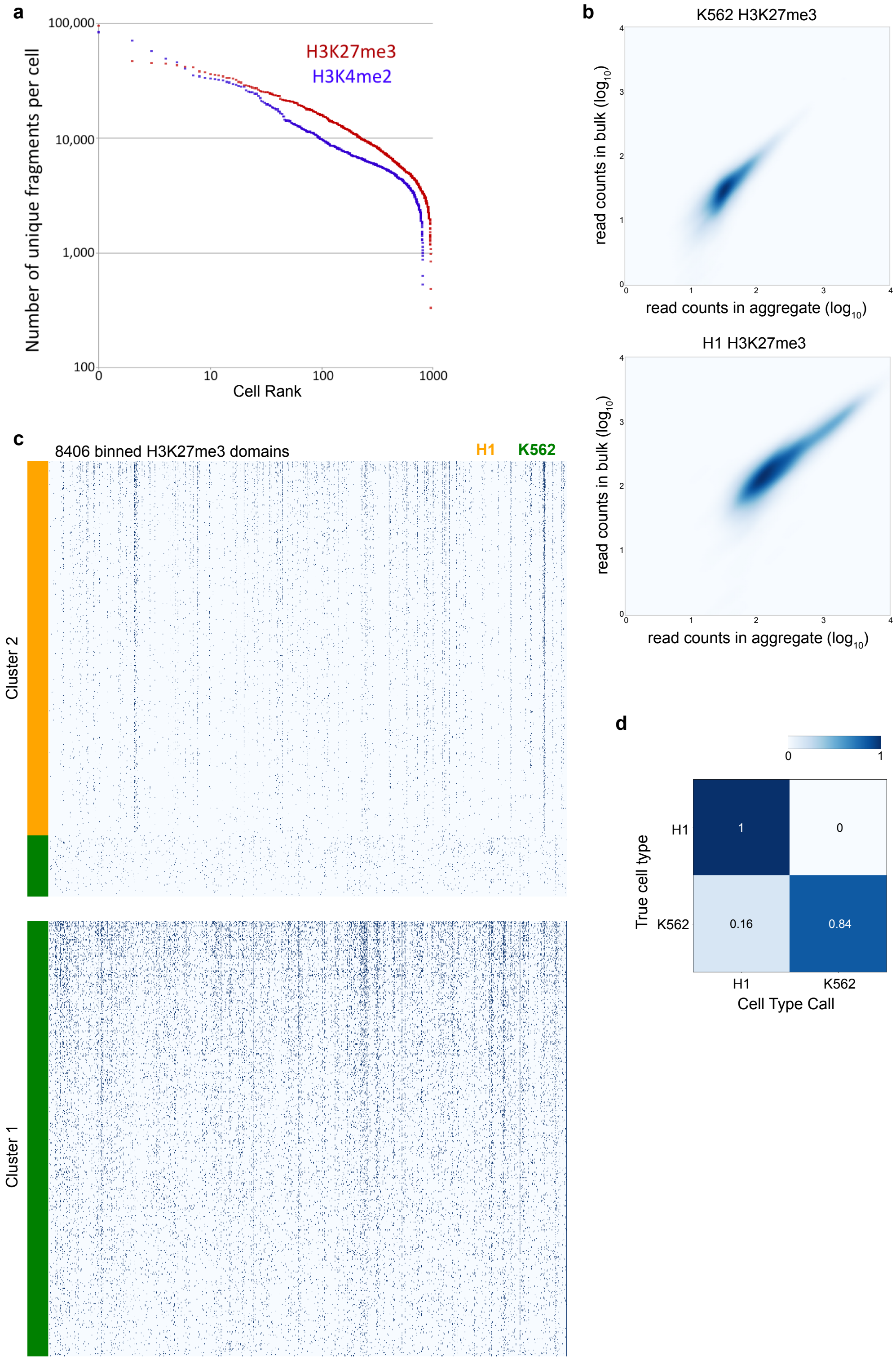

Supplementary Figure 6. Single-cell CUT&Tag fragment recovery.
(a) Plot of the number of unique reads from each single cell after scCUT&Tag. 956 single K562 cells in H3K27me3 scCUT&Tag and 808 single K562 cells in H3K4me2 scCUT&Tag. Cells were ranked based on their number of unique reads.
(b) Correlation of genomic profiles for bulk CUT&Tag and aggregated scCUT&Tag experiments with K562 and H1 cells. The R-value for K562 cells is 0.85, and that for H1 cells is 0.88.
(c) Clusters of single cells based on H3K27me3 profiles. Reads from 479 H1 and 479 K562 cells were binned into a combined list of H3K27me3 domains called from bulk profiles and sorted by Kmeans with k=2. Cells are color-coded on the left end of the matrix. All H1 single cells are assigned to one cluster, while 84% of K562 single cell profiles are assigned to the other cluster. The 78 mis-assigned K562 profiles have very low coverage.
(d) Confusion matrix of H3K27me3 scCUT&Tag profile sorting in Supplementary Figure 6c.

Supplementary Information

Supplementary Note 1: Data processing for all Figures in Kaya-Okur *et al* (2019).

Peak calling:

In all cases except as noted MACS2 was used to call peaks on bedfiles for CUT&RUN and CUT&Tag,datasets with no input files with the following flags:

macs2 callpeak -t input_BED -f BEDPE -p 1e-5 --keep-dup all -n output_prefix

Heatmapping:

In all cases the midpoints of peak regions were used as the center in +/- 1kb regions with 50 bp bins were generated, ordered by mean signal and plotted using deepTools. The display range was adjusted to the maximum and minimum signals for each heatmap, and the number of regions is reported in the figure.

New data:

All sequencing data is deposited in GEO under the super-entry GSE124690, including the bulk GSE124557 and the single-cell entries GSE124680 and GSE124683.

Software packages used:

MACS2 version 2.1.1, deepTools version 3.0.2, seaborn version 0.8.1, and FIMO version 5.0.4.

For Figure 2a:

UCSC Genome Browser tracks for datasets (GSM788088, GSM3391661, GSM3536515, GSM3536516, GSM3536517, GSM3536520, GSM3560264, GSE99173) from K562 cells were plotted with autoscaling, except for the IgG control, which was set to a track height of 20.

For Figure 2b:

UCSC Genome Browser tracks for datasets (GSM733748, GSM3536498, GSM3536499, GSM3536500, GSM3536502, GSM3560256) from H1 cells were plotted with autoscaling, except for the IgG control, which was set to a track height of 20.

For Figure 2c:

K4me1 bedfiles for CUT&RUN (GSM3391662) and for CUT&Tag (GSM3536516) were used for heatmapping. For H3K4me1 ChIP-seq, we used the list of peaks associated with the GEO entry GSM733692.

For Figure 2d:
Bedfiles for RNAPII CUT&Tag (GSM3391654) were used for heatmapping. Promoters were ordered by gene expression determined by RNA-seq (GSM207236). Promoters within 1 kb of each other were excluded. This promoter list is provided as Source Data.

For Figure 2e:
Peaks for RNAPII-S5p CUT&Tag were called using MACS2 on the HsSc_PolS5_F_0802, HsSc_PolS5_T_0802 dataset. PRO-seq data (SRA GSM1480327) for K562 cells were clustered using a custom script described in Ref. 10. Heat maps were displayed using Java Treeview (http://jtreeview.sourceforge.net/).

For Figure 2f:
Bedfiles for ATAC-seq (GSM2695560) and for H3K4me2 CUT&RUN (GSM3391663) were used for peak calling. For ATAC calls, we performed a tail truncation removing the 2.5% most significant and least significant peaks to limit sites enriched for PCR duplicates or with very low read counts. Heat maps were then plotted.

For Figure 2g:
We down sampled CUT&RUN (GSM3391662), CUT&Tag (GSM3536516), ChIP-seq (GSM733692) data on H3K4me1 to ~8M reads. We then took the top 10,000 ChIP-seq (GSM733692) Peaks for K4me1 (provided as Source Data) with the highest signal and plotted the signal of all three datasets at those sites with the command plotProfile from DeepTools. We noticed ChIP-seq had relatively low signal at those sites, so we plotted ChIP-seq (GSM733692) data at full sequencing depth as well which was ~29M reads.

For Figure 2h:
We took 53,805 accessible sites determined by ATAC-seq (GSM2695560), 53,805 random genomic sites and plotted the profile of ATAC-seq signal and CUT&TAG signal for H3K4me2 (GSE124557) at those sites using the plotProfile tool from deeptools. CUT&TAG data for

H3K4me2 was down sampled to the same depth as ATAC-seq. The list of accessible sites is provided as Source Data.

For Figure 3a:

We divided the genome into 500 bp bins and reads in each bin from H3K4me1 for ChIP-seq (GSM733692), CUT&RUN (GSM3391662), and CUT&Tag (GSM3536516) experiments along with CUT&Tag for IgG (GSM3560264) were summed. Pearson correlations were generated by comparing between all pairs of the log transformed read count array. These Pearson correlations were then used for hierarchical clustering and the figure was generated using the seaborn package on Python.

For Figure 3b:

We subsampled each of the datasets to 20M, 10M, 5M, 2.5M, 1M, and 500K mapped reads, and then called peaks on each set. For ChIP-seq and ATAC-seq datasets, we changed the flag -f to BED instead of BEDPE because these datasets were single-end reads. The sets of peaks for each subsampled population of reads are provided as Source Data. We then binned the reads in a +/- interval around peaks and calculated the percent of reads in peaks of the total read count.

For Figure 4a:

IGV Genome Browser track for NPAT CUT&Tag data (GSM3536519) for chromosomes 1 and 6 was plotted with a fixed scale of the maximum of 19863.

For Figure 4b:

To determine read counts at histone gene promoters, we binned read counts within a +/- 100 bp region of each histone gene in a NPAT CUT&Tag experiment (GSM3536519). To determine read counts at hypersensitive sites, we repeated binning and counting for all ATAC-seq peaks that do not overlap with a histone gene promoter. To determine background, we selected 10,000 positions in the genome at random and counted reads at those sites. These read counts are plotted in the histogram.

For Figure 4c:

We curated a list of replication-dependent histone genes and counted NPAT CUT&Tag reads mapping around +/- 100 bp of the summit of their promoters. We generated a list of “ATAC”

sites as peak calls from ATAC-seq data, and then removed histone gene promoters from that list. We then counted reads that mapped +/- 100 bp of the summit of these “ATAC-only” sites, and generated a histogram to display the results.

For Figure 5a:

We obtained the MA0139.1 CTCF motif for humans from the JASPAR database. We used FIMO (http://meme-suite.org/doc/fimo.html) with default parameters to search the hg19 genome for matches to the CTCF motif, and ordered sites by their p-value. Read counts from CTCF ChIP-seq (GSM733719), CUT&RUN (GSM3391659), and CUT&Tag (GSM3560258) experiments on this list of sites were heatmapped.

For Figure 5b:

We generated a list of CTCF-bound sites by calling peaks on CTCF CUT&RUN data (GSM3391659) and counted CTCF CUT&Tag reads mapping around +/- 100 bp of the summit of those sites. We generated a list of “ATAC” sites as peak calls from ATAC-seq data, and then removed CTCF-bound sites from that list. We then counted reads that mapped +/- 100 bp of the summit of these “ATAC-only” sites and generated a histogram to display the results.

For Figure 5c:

We used the list of CTCF motifs from Figure 5a and mapped read ends centered on these motifs for each salt condition (GSM3560257, GSM3560258, and GSM3560259). We then smoothed mapped read ends along a 11bp window using a Savitzky-Golay filter (SciPy) with a polynomial order of zero.

For Figure 5d:

UCSC Genome Browser tracks for CTCF CUT&Tag (CTCF_5m_1), ATAC (ATAC_5m_1), H3K4me2 ChIP-seq (ENCODE_5m_1), H3K4me2 CUT&Tag (H3K4me2_5m_1), H3K4me3 CUT&Tag (H3K4me2_5m_2), H3K4me1 CUT&Tag (H3K4me1_5m_1), H2A.Z CUT&Tag (H2AZ_5m) were plotted with autoscaling.

For Figure 6b-c:

IGV Genome Browser tracks for 908 individual single cells profiled by H3K27me3 scCUT&Tag (GSM124680) and for 807 individual single cells profiled by H3K4me2 scCUT&Tag (GSM124683) were displayed showing individual fragments.

For Figure 6d:

We ran MACS2 on bulk data for H3K4me2 CUT&TAG data (GSE124557) with default settings. For each H3K4me2 single cell (GSM124683) we counted the number of reads that fell within those narrow peaks and calculated the fraction of reads in peaks to the total number of reads for each single cell.

For Figure 6e:

We ran SEACR on bulk H3K27me3 data for K562 (GSM3536515) and H1 (GSM3536498) cells relative to an IgG control to define H3K27me3 enriched domains. We then counted the number of reads that fell within those domains and calculated the fraction of reads in domains per single cell for histone mark H3K27me3 in H1 and K562 (GSM124680). List of domains for each K562 and H1 is provided as Source Data.

For Figure 6f:

IGV Genome Browser tracks for 479 individual H1 and 479 individual K562 single cells profiled by H3K27me3 scCUT&Tag (ID and ID) were displayed showing individual fragments.

For Supplementary Figure 1a:

Read counts mapping to the hg19 and *E. coli* genomes from a serial dilution of starting cells (HK_Hs_K5II_K27me?_*_0912) is plotted.

For Supplementary Figure 1b:

IGV Genome Browser tracks for H3K27me3 CUT&Tag using different starting cell numbers (HK_Hs_K5II_K27me5_6k_0912.bed, HK_Hs_K5II_K27me7_600_0912.bed, and HK_Hs_K5II_K27me9_60_0912.bed) was plotted with autoscaling.

For Supplementary Figure 2a:

UCSC Genome Browser tracks for (GSM3536521, GSM3536522, GSM3536523, GSM3536518, GSM3536514) were plotted with autoscaling.

For Supplementary Figure 2b:

UCSC Genome Browser tracks for (GSM3536501, GSM3536497, GSM3536506) were plotted with autoscaling.

For Supplementary Figure 2c:

We divided the genome into 500 bp bins and reads in each bin from H3K27me3 (GSE31755) for ChIP-seq (GSM733692), CUT&RUN (GSM3391661) and CUT&Tag (GSM3536515) experiments were summed. Pearson correlations were generated by comparing between all pairs of the log transformed read count array. These Pearson correlations were then used for hierarchical clustering and the figure was generated using the seaborn package on Python.

For Supplementary Figure 3a:

UCSC Genome Browser tracks for (GSM3536521, GSM3536522, GSM3536523, GSM3536518) were plotted with autoscaling.

For Supplementary Figure 3b:

Bedfiles for RNAPII CUT&Tag (GSM3536521, GSM3536522, GSM3536523) were used for heat mapping. Promoters were ordered by gene expression determined by RNA-seq (GSM207236). Promoters within 1 kb of each other were excluded. This promoter list is provided as Source Data.

For Supplementary Figure 3c:

We ordered gene promoters by RNA-seq (Source Data) and plotted read counts on (+) and (–) strands from RNAPII S2p (GSM3536521), RNAPII S5p (GSM3536522), and RNAPII S7p (GSM3536523) CUT&TAG experiments. Strands were recolored in Photoshop and levels were adjusted to maximize display intensity.

For Supplementary Figure 4:

We used MACS2 to call peaks on pooled NPAT CUT&Tag data (SH_Hs_NPA1_20190226… SH_Hs_NPA4_20190226, SH_Hs_NPB1_20190226… SH_Hs_NPB4_20190226), and ranked them by their maximum read count. Sites falling within histone gene clusters were colored blue, and other sites are colored orange.

For Supplementary Figure 5a:

Library profiles after CUT&Tag experiments were measured using an Agilent Tapestation 4200 with D1000 High Sensitivity Screen tapes.

For Supplementary Figure 5b:

IGV Genome Browser track for CUT&Tag with the following datasets were plotted with fixed scaling: SH_Hs_NPA1_20190226, SH_Hs_NPA2_20190226, SH_Hs_NPA4_20190226, SH_Hs_NPA8_20190226, SH_Hs_NPB1_20190226, SH_Hs_NPB2_20190226, SH_Hs_NPB4_20190226, SH_Hs_NPB8_20190226, SH_Hs_me3A1_20190226, SH_Hs_me3A2_20190226, SH_Hs_me3A4_20190226,SH_Hs_me3A8_20190226, SH_Hs_me3B1_20190226, SH_Hs_me3B2_20190226, SH_Hs_me3B4_20190226, and SH_Hs_me3B8_20190226.

For Supplementary Figure 5c:

Mapped reads to the human and *E. coli* genomes for each sample in Figure 5b.

For Supplementary Figure 6a:

Unique mapped read counts for 908 individual single cells profiled by H3K27me3 scCUT&Tag (GSM124680) and for 807 individual single cells profiled by H3K4me2 scCUT&Tag (GSM124683) were graphed.

For Supplementary Figure 6b:

Bulk and Aggregate profiles for both H1 (GSM3536498, Single Cells: GSE124690) and K562 (GSM3536515, Single Cells: GSE124690) histone mark, H3K27me3, (Duplicates were not removed) were binned into domains. Counts in domains were normalized by total number of reads and multiplied by 10^6, and then plotted with a $\log_{10}$ scale. The list of domains is provided as Source Data.

For Supplementary Figure 6c:

We generated a master list 8406 domains in K562 and H1 cells by first intersecting H1 and K562 domains and generating a "shared" list of domains. We then removed the list of shared sites from the H1 and K562 specific list and merged the K562, H1, and shared list of domains so that there would be no redundant sites. The master list of domains is provided as Source Data. We then generated a binary matrix where each row is a single cell and each column is a domain. A 1 indicates the presence of a read within a domain and a 0 that it did not. 15 domains defined in bulk data were not populated by any reads in single cells and were removed from the

analysis. We then ran Kmeans clustering for k=2 using the scikit package from python and plotted the resulting clusters as heatmaps using seaborn.

For Supplementary Figure 6d:

The scikit package was used to generate a confusion matrix of the Kmeans clustering results from supplementary figure 6c.

References

deepTools:

Ramírez, F. *et al.* deepTools2: a next generation web server for deep-sequencing data analysis. *Nucleic Acids Research* **44**, W160-W165 (2016).

MACS2:

"Zhang, Y. *et al*. Model-based Analysis of ChIP-Seq (MACS). *Genome Biology* **9**, R137 (2008).

FIMO:

Grant, C.E. *et al*. FIMO: scanning for occurrences of a given motif. *Bioinformatics **27***, 1017-1018 (2011).

JASPAR:

Khan, A. *et al*. JASPAR 2018: update of the open-access database of transcription factor binding profiles and its web framework. *Nucleic Acids Research* **46**, D260–D266 (2018).

RNA-seq data:

ENCODE Project Consortium. An integrated encyclopedia of DNA elements in the human genome. *Nature* **489**, 57-74 (2012).

Scikit:

Pedregosa *et al.* Scikit-learn: Machine Learning in Python. JMLR **12**, 2825-2830 (2011).

SEACR:

Meers, Michael P *et al.* A streamlined protocol and analysis pipeline for CUT&RUN chromatin profiling. *BioRxiv* 569129 (2019)
