# Supplementary material for: CUT&Tag for efficient epigenomic profiling of small samples and single cells
Source: Nat Commun. 2019 Apr 29;10:1930. doi: 10.1038/s41467-019-09982-5 (PMC6488672; doi:10.1038/s41467-019-09982-5)
Supplement: Supplementary file 3 — Reporting Summary [file 41467_2019_9982_MOESM3_ESM.pdf]

nature research

Corresponding author(s): Steven Henikoff

Last updated by author(s): Apr 5, 2019

Reporting Summary

Nature Research wishes to improve the reproducibility of the work that we publish. This form provides structure for consistency and transparency in reporting. For further information on Nature Research policies, see Authors & Referees and the Editorial Policy Checklist.

Statistics

For all statistical analyses, confirm that the following items are present in the figure legend, table legend, main text, or Methods section.

n/a	Confirmed	
☐	☒	The exact sample size (*n*) for each experimental group/condition, given as a discrete number and unit of measurement
☐	☒	A statement on whether measurements were taken from distinct samples or whether the same sample was measured repeatedly
☒	☐	The statistical test(s) used AND whether they are one- or two-sided<br>*Only common tests should be described solely by name; describe more complex techniques in the Methods section.*
☒	☐	A description of all covariates tested
☐	☒	A description of any assumptions or corrections, such as tests of normality and adjustment for multiple comparisons
☐	☒	A full description of the statistical parameters including central tendency (e.g. means) or other basic estimates (e.g. regression coefficient) AND variation (e.g. standard deviation) or associated estimates of uncertainty (e.g. confidence intervals)
☒	☐	For null hypothesis testing, the test statistic (e.g. *F*, *t*, *r*) with confidence intervals, effect sizes, degrees of freedom and *P* value noted<br>*Give P values as exact values whenever suitable.*
☒	☐	For Bayesian analysis, information on the choice of priors and Markov chain Monte Carlo settings
☒	☐	For hierarchical and complex designs, identification of the appropriate level for tests and full reporting of outcomes
☐	☒	Estimates of effect sizes (e.g. Cohen's *d*, Pearson's *r*), indicating how they were calculated

Our web collection on statistics for biologists contains articles on many of the points above.

Software and code

Policy information about availability of computer code

Data collection	Detailed in the manuscript
Data analysis	Detailed in the manuscript

For manuscripts utilizing custom algorithms or software that are central to the research but not yet described in published literature, software must be made available to editors/reviewers. We strongly encourage code deposition in a community repository (e.g. GitHub). See the Nature Research guidelines for submitting code & software for further information.

Data

Policy information about availability of data

All manuscripts must include a data availability statement. This statement should provide the following information, where applicable:

- Accession codes, unique identifiers, or web links for publicly available datasets
- A list of figures that have associated raw data
- A description of any restrictions on data availability

Data availability. All sequencing data has been deposited in GEO, and will be made public upon acceptance.

For review, the following link allows reviewer access to all data files at GEO: https://www.ncbi.nlm.nih.gov/geo/query/acc.cgi?acc=GSE124557
enter token mxmpcimsldyvzmp into the box.

Field-specific reporting

Please select the one below that is the best fit for your research. If you are not sure, read the appropriate sections before making your selection.

☒ Life sciences ☐ Behavioural & social sciences ☐ Ecological, evolutionary & environmental sciences

For a reference copy of the document with all sections, see nature.com/documents/nr-reporting-summary-flat.pdf

Life sciences study design

All studies must disclose on these points even when the disclosure is negative.

Sample size	n/a
Data exclusions	Sequencing reads mapping to the mitochondrial genome were removed from all datasets.
Replication	At least 2 biological replicates were profiled.
Randomization	n/a
Blinding	n/a

Reporting for specific materials, systems and methods

We require information from authors about some types of materials, experimental systems and methods used in many studies. Here, indicate whether each material, system or method listed is relevant to your study. If you are not sure if a list item applies to your research, read the appropriate section before selecting a response.

Materials & experimental systems

n/a	Involved in the study	
☐	☒	Antibodies
☐	☒	Eukaryotic cell lines
☒	☐	Palaeontology
☒	☐	Animals and other organisms
☒	☐	Human research participants
☒	☐	Clinical data

Methods

n/a	Involved in the study	
☐	☒	ChIP-seq
☒	☐	Flow cytometry
☒	☐	MRI-based neuroimaging

Antibodies

Antibodies used	All antibodies and suppliers are listed in the Methods: Biological materials section
Validation	All antibodies are commercially available, and have been verified by Western blotting or by peptide ELISA described on the manufacturer's specification sheets.

Eukaryotic cell lines

Policy information about cell lines

Cell line source(s)	K562: ATCC, catalog# CCL-243. H1: WiCell WA01-WB35186.
Authentication	none were authenticated.
Mycoplasma contamination	Negative for mycoplasma
Commonly misidentified lines (See ICLAC register)	n/a

ChIP-seq

Data deposition

☒ Confirm that both raw and final processed data have been deposited in a public database such as GEO.

☒ Confirm that you have deposited or provided access to graph files (e.g. BED files) for the called peaks.

Data access links<br>*May remain private before publication.*	Reviewer access link: https://www.ncbi.nlm.nih.gov/geo/query/acc.cgi?acc=GSE124557<br>then type token mxmpcimsldyvzmp into the box.
Files in database submission	GSM3536497 H1_K27ac_(20180919_HK_Hs_H1I_K27ac_0912)<br>GSM3536498 H1_K27me3_(20180919_HK_Hs_H1I_K27me3_0912)<br>GSM3536499 H1_K4me1_(20180919_HK_Hs_H1I_K4me1_0912)<br>GSM3536500 H1_K4me2_(20180919_HK_Hs_H1I_K4me2_0912)<br>GSM3536501 H1_K4me3_(20180919_HK_Hs_H1I_K4me3_0912)<br>GSM3536502 H1_PolSer25P_(20180919_HK_Hs_H1I_PolSer25P_0912)<br>GSM3536503 H1_PolSer2P_(20180919_HK_Hs_H1I_PolSer2P_0912)<br>GSM3536504 H1_PolSer5P_(20180919_HK_Hs_H1I_PolSer5P_0912)<br>GSM3536505 H1_PolSer7P_(20180919_HK_Hs_H1I_PolSer7P_0912)<br>GSM3536506 H1_Sox2_(20180919_HK_Hs_H1I_Sox2_0912)<br>GSM3536507 K562_H3K27me3_200_(20180919_HK_Hs_K5II_K27me8_200_0912)<br>GSM3536508 K562_H3K27me3_20k_(20180919_HK_Hs_K5II_K27me4_20k_0912)<br>GSM3536509 K562_H3K27me3_2k_(20180919_HK_Hs_K5II_K27me6_2k_0912)<br>GSM3536510 K562_H3K27me3_600_(20180919_HK_Hs_K5II_K27me7_600_0912)<br>GSM3536511 K562_H3K27me3_60k_(20180919_HK_Hs_K5II_K27me3_60k_0912)<br>GSM3536512 K562_H3K27me3_60_(20180919_HK_Hs_K5II_K27me9_60_0912)<br>GSM3536513 K562_H3K27me3_6k_(20180919_HK_Hs_K5II_K27me5_6k_0912)<br>GSM3536514 K562_K27ac_(20180919_HK_Hs_K5I_K27ac_0912)<br>GSM3536515 K562_K27me3_(20180919_HK_Hs_K5I_K27me3_0912)<br>GSM3536516 K562_K4me1_(20180919_HK_Hs_K5I_K4me1_0912)<br>GSM3536517 K562_K4me2_(20180919_HK_Hs_K5I_K4me2_0912)<br>GSM3536518 K562_K4me3_(20180919_HK_Hs_K5I_K4me3_0912)<br>GSM3536519 K562_NPAT_(20181120_HK_Hs_K5I_NPAT_0912)<br>GSM3536520 K562_PolSer25P_(20180919_HK_Hs_K5I_PolSer25P_0912)<br>GSM3536521 K562_PolSer2P_(20180919_HK_Hs_K5I_PolSer2P_0912)<br>GSM3536522 K562_PolSer5P_(20180919_HK_Hs_K5I_PolSer5P_0912)<br>GSM3536523 K562_PolSer7P_(20180919_HK_Hs_K5I_PolSer7P_0912)<br>GSM3560256 H1_IgG_(20180919_HK_Hs_H1_IgG_0912)<br>GSM3560257 K562_CTCF_150mM_(20190104_SH_Hs_C15_20181224,6)<br>GSM3560258 K562_CTCF_300mM_(20190104_SH_Hs_C30_20181224,6)<br>GSM3560259 K562_CTCF_500mM_(20190104_SH_Hs_C50_20181224,6)<br>GSM3560260 K562_H2AZ_(20180919_HK_Hs_K5_H2AZ_0912)<br>GSM3560261 K562_H3K27me3_(20190104_SH_Hs_M30_20181224)<br>GSM3560262 K562_H3K9ac_(20190104_SH_Hs_H91,2_181227)<br>GSM3560263 K562_H4Kac_(20190104_SH_Hs_H41_20181227)<br>GSM3560264 K562_IgG_(20180919_HK_Hs_K5_IgG_0912)<br>GSM3560265 K562_NPAT_(20180919_HK_Hs_K5_NPAT_0912)<br>GSM3680214 K562_CTCF_(20180919_HK_Hs_K5_CTCF_0912)<br>GSM3680215 K562_H3K27me3_CST9733_10d-RT_1000th_(20190227_SH_Hs_me3B8_20190217)<br>GSM3680216 K562_H3K27me3_CST9733_10d-RT_125th_(20190227_SH_Hs_me3B1_20190217)<br>GSM3680217 K562_H3K27me3_CST9733_10d-RT_250th_(20190227_SH_Hs_me3B2_20190217)<br>GSM3680218 K562_H3K27me3_CST9733_10d-RT_500th_(20190227_SH_Hs_me3B4_20190217)<br>GSM3680219 K562_H3K27me3_CST9733_fresh_1000th_(20190227_SH_Hs_me3A8_20190217)<br>GSM3680220 K562_H3K27me3_CST9733_fresh_125th_(20190227_SH_Hs_me3A1_20190217)<br>GSM3680221 K562_H3K27me3_CST9733_fresh_250th_(20190227_SH_Hs_me3A2_20190217)<br>GSM3680222 K562_H3K27me3_CST9733_fresh_500th_(20190227_SH_Hs_me3A4_20190217)<br>GSM3680223 K562_H3K4me1_Abcam_8895_(20180810_SH_Hs_H3K4me1_0806)<br>GSM3680224 K562_H3K4me1_ActMot_39113_(20180817_SH_Hs_7_H2AZ_0816)<br>GSM3680225 K562_H3K4me2_Upstate_07-030_(20180817_SH_Hs_11_K4me2_0816)<br>GSM3680226 K562_H3K4me3_Abcam_ab8580_(20180817_SH_Hs_12_K4me3_0816)<br>GSM3680227 K562_IgG_ABIN101961_(20190104_SH_Hs_IgG_20181224)<br>GSM3680228 K562_NPAT_Thermo-PA5-66839_10d-RT_1000th_(20190227_SH_Hs_NPB8_20190217)<br>GSM3680229 K562_NPAT_Thermo-PA5-66839_10d-RT_125th_(20190227_SH_Hs_NPB1_20190217)<br>GSM3680230 K562_NPAT_Thermo-PA5-66839_10d-RT_250th_(20190227_SH_Hs_NPB2_20190217)<br>GSM3680231 K562_NPAT_Thermo-PA5-66839_10d-RT_500th_(20190227_SH_Hs_NPB4_20190217)<br>GSM3680232 K562_NPAT_Thermo-PA5-66839_fresh_1000th_(20190227_SH_Hs_NPA8_20190217)<br>GSM3680233 K562_NPAT_Thermo-PA5-66839_fresh_125th_(20190227_SH_Hs_NPA1_20190217)<br>GSM3680234 K562_NPAT_Thermo-PA5-66839_fresh_250th_(20190227_SH_Hs_NPA2_20190217)<br>GSM3680235 K562_NPAT_Thermo-PA5-66839_fresh_500th_(20190227_SH_Hs_NPA4_20190217)
Genome browser session<br>(e.g. UCSC)	n/a

Methodology

Replicates	At least 2 replicates were performed. Comparative analysis is the topic of this manuscript.
Sequencing depth	All Experiments were paired-end. Sequencing depths and sampling is reported in the manuscript.
Antibodies	All antibodies and sources are provided in the Methods section.
Peak calling parameters	Parameters are specified in the Methods section.
Data quality	Data quality assessment is the topic of this manuscript, and is reported.
Software	A link to custom code is provided in the Methods section.
